# Acceptance-Based Pre-Exposure Prophylaxis Intervention (ACTPrEP) to Engage Young Black Men Who Have Sex With Men in the Southern United States: Protocol for a Pilot Randomized Controlled Trial

**DOI:** 10.2196/65921

**Published:** 2025-06-20

**Authors:** Trisha Arnold, Kayla K Giorlando, Andrew P Barnett, Brandon A Gaudiano, Cara M Antonaccio, A Rani Elwy, Precious Patrick Edet, Lori M Ward, Laura Whiteley, Sarah Bailey, Brooke G Rogers, Avery Leigland, Jack C Rusley, Larry K Brown

**Affiliations:** 1 Department of Psychiatry Rhode Island Hospital Providence, RI United States; 2 Department of Psychiatry and Human Behavior Warren Alpert Medical School of Brown University Providence, RI United States; 3 Center for Healthcare Organization and Implementation Research VA Bedford Healthcare System Bedford, MA United States; 4 Departments of Nursing, Medicine, and Population Health Sciences University of Mississippi Medical Center Jackson, MS United States; 5 Department of Psychiatry Boston Medical Center Boston, MA United States

**Keywords:** HIV prevention, pre-exposure prophylaxis, young Black men who have sex with men, acceptance and commitment therapy, protocol

## Abstract

**Background:**

Given the high rates of HIV infection among young Black men who have sex with men in Jackson, Mississippi, and the underuse of pre-exposure prophylaxis (PrEP) in this population, there is a critical need for innovative interventions to promote PrEP use. Acceptance and commitment therapy (ACT) is a promising intervention for health behavior change.

**Objective:**

This study aims to develop and test an intervention, ACTPrEP, to increase PrEP use among young Black men who have sex with men.

**Methods:**

We conducted in-depth interviews with 20 young Black men who have sex with men and 10 PrEP staff to inform the development of ACTPrEP, an intervention that uses ACT to improve PrEP use. Specific ACTPrEP adaptations from the qualitative interviews included service settings (interventionists should be PrEP informed and well-integrated), target audiences (provide check-in calls, be caring, and authentically relate), modes of delivery (flexible delivery), and cultural adaptation (normalize PrEP use). A pilot randomized controlled trial will evaluate the feasibility and acceptability of ACTPrEP compared to that of Enhanced Standard of Care in promoting PrEP use among young Black men who have sex with men (N=66). We will specifically evaluate group differences in the proportion of participants who initiate PrEP, PrEP adherence, and PrEP persistence. Measures will be collected at baseline, 6 weeks, and 12 weeks.

**Results:**

Research activities began in September 2023 and will be ongoing through June 2026. Preliminary findings are anticipated to be available in late 2026.

**Conclusions:**

This study will inform the development of a larger randomized controlled trial to evaluate the efficacy of ACTPrEP for increasing PrEP use among young Black men who have sex with men.

**Trial Registration:**

ClinicalTrials.gov NCT05087680; https://clinicaltrials.gov/study/NCT05087680

**International Registered Report Identifier (IRRID):**

DERR1-10.2196/65921

## Introduction

The HIV epidemic disproportionately affects men who have sex with men, with a significant burden borne by young Black men who have sex with men [1]. In the United States, Black men who have sex with men account for a quarter of new HIV diagnoses despite representing less than 1% of the population [2]. Young Black men who have sex with men are also substantially more likely to receive an HIV diagnosis in their lifetime compared to their White counterparts [1]. In the South, the burden is particularly high. Jackson, Mississippi, experiences some of the highest rates of HIV infection among urban men who have sex with men [3], with a majority occurring in young Black men who have sex with men [4].

Pre-exposure prophylaxis (PrEP) is a highly effective HIV prevention strategy involving taking antiretroviral medication to prevent HIV acquisition before potential exposure [5,6]. Studies have demonstrated the high efficacy of PrEP when taken as prescribed [7]. Despite this, significant disparities exist in PrEP uptake and adherence, particularly among young Black men who have sex with men [8]. In 2021, the PrEP to need ratio was lowest among Black individuals aged 13-24 years when compared to other races and age groups [9]. While there has been a 34% decrease in new HIV infections among young men who have sex with men, decreases are not equitable among ethnic groups [10]. Young Black men who have sex with men had a 27% decrease, while young White men who have sex with men had a 45% decrease [10]. There have also been multiple studies that have identified barriers preventing young Black men who have sex with men from accessing and using PrEP, including lack of priority, lack of perceived risk, lack of PrEP knowledge, stigma and fear, misunderstood side effects, and payment for PrEP [11-13]. These highlight the critical need for interventions to address the underuse of PrEP among young Black men who have sex with men.

Acceptance and commitment therapy (ACT) is a well-established psychological intervention that has been widely used to facilitate health behavior change, particularly in populations experiencing emotional and psychological barriers to medical adherence [14]. ACT targets psychological inflexibility, which refers to the tendency to avoid or suppress thoughts, emotions, and bodily sensations even when doing so interferes with valued actions and health-related goals [14]. Psychological inflexibility is a known barrier to health behavior engagement, including medication adherence, preventive care, and chronic disease management [15]. Studies have linked avoidance behaviors, delayed medical care, and nonadherence to prescribed treatments with poor health outcomes in multiple chronic conditions [16-20]. ACT has been successfully applied across various health domains to improve adherence and promote sustained health behavior change. Research shows that ACT-based interventions improved smoking cessation and substance use reduction through enhanced psychological flexibility [21], medication adherence and pain management in chronic disease populations [22,23], diet and physical activity improvements among patients with cardiovascular disease [24], weight management and disordered eating behaviors [25], symptom reduction in individuals with severe health anxiety [26], and self-management behaviors in diabetes and other chronic conditions using digital ACT-based interventions [27].

Given ACT’s efficacy in improving health-related behaviors, it presents a promising intervention for addressing psychological barriers to PrEP use. ACT can help individuals identify personal values regarding various domains of life (ie, their own health), as well as how taking PrEP can contribute to living by those personal values [14]. By promoting psychological flexibility, ACT can help individuals identify and address avoidant internal experiences that might hinder PrEP use, such as anxieties or negative beliefs about taking medication, and help them approach these experiences in service of living according to their personal values. Furthermore, ACT interventions can be delivered effectively in a variety of formats, including brief sessions, and anyone can be trained to deliver the intervention, making it both adaptable and feasible for reaching young Black men who have sex with men in diverse settings [28].

Given the high rates of HIV infection among young Black men who have sex with men in Jackson, Mississippi, and the underuse of PrEP in this population, there is a critical need for innovative interventions to promote PrEP use. ACT’s potential to address psychological barriers to PrEP use and its adaptability for community-based settings make it a promising avenue for investigation [28]. This pilot randomized controlled trial (RCT) aims to assess the feasibility and acceptability of an ACT-based intervention for increasing PrEP use (ACTPrEP) among young Black men who have sex with men in Jackson, Mississippi, in the United States.

## Methods

### Study Design

This study will be conducted in 2 phases. The first phase, which has been completed, consisted of developmental interviews with 20 young Black men who have sex with men and 10 clinic staff. These in-depth interviews, completed by 2 researchers trained in conducting qualitative interviews (TA and APB), assessed quantitative measures, intervention design, and experiences relevant to PrEP engagement. This helped researchers select and refine components of the ACTPrEP intervention. The second phase will be a single-site, pilot RCT conducted at the University of Mississippi Medical Center STI/HIV Testing Clinic. RCT participants (N=66) will be assigned to either the ACTPrEP intervention or the Enhanced Standard of Care (ESOC) arm using a random allocation program. The allocation ratio will be 1:1, with 33 participants assigned to each arm.

### Participants

Staff participants (developmental phase only) are nurses, PrEP navigators, and PrEP prescribers who work directly with PrEP-eligible Black men who have sex with men.

Criteria for young Black men who have sex with men participants (for both study phases) include being aged between 18 and 34 years old, able to speak in English, being HIV-negative, male sex having been assigned at birth (including transgender women), report having sex with a man in the past 3 months, being PrEP-eligible according to Centers for Disease Control and Prevention guidelines, not having taken PrEP in the past 3 months, and not currently being enrolled in another PrEP-related study. There will not be an overlap between those enrolled in the developmental phase and the pilot RCT. The inclusion criteria were updated in April 2024 for the pilot RCT to include individuals not currently taking PrEP and individuals who are currently taking PrEP. Since ACTPrEP focuses on both PrEP update and PrEP persistence, young Black men who have sex with men who are taking PrEP or qualify for a prescription benefit from the study. The change in inclusion criteria decision was also made as an effort to enhance recruitment.

### Developmental Phase: Qualitative Interviews

#### Procedures

Participants were recruited through word of mouth. Specifically, young Black men who have sex with men were informed about the study by the clinic staff during clinic visits, and clinic staff were informed about the study by research staff. Those interested were screened for eligibility by the research assistant. Participants completed informed consent via the HIPAA (Health Insurance Portability and Accountability Act)-compliant, electronic signature software developed by DocuSign, Inc, and were then scheduled to complete an interview. Developmental interviews were conducted remotely via a HIPAA-compliant videoconferencing platform, Zoom (Zoom Video Communications). Each interview lasted approximately 45-60 minutes and was conducted by 2 researchers trained in conducting qualitative interviews (TA and APB). Participants were also asked to complete a brief web-based survey using the HIPAA-compliant data collection platform, REDCap (Research Electronic Data Capture, Vanderbilt University) [29], which took roughly 10-15 minutes. The survey queried about PrEP structural barriers, PrEP stigma, and psychological flexibility. Participants could complete the survey from their personal electronic devices or on a computer in a private area of the University of Mississippi Medical Center.

#### Interviews

Staff interviews focused on the motives and perceived values young Black men who have sex with men have to be engaged in PrEP care and assessed the acceptability of the ACTPrEP design. Interviews with young Black men who have sex with men examined the impact of ACT processes that influence PrEP engagement in care: awareness of experiences, acceptance of experiences, and values. Participants were probed to discuss values within different areas of life (eg, recreational, spiritual, career, family, and health) and then asked how PrEP use aligned with those values. If young Black men who have sex with men indicated no prior PrEP knowledge, they were provided this brief education: “PrEP is a pill you can take once a day to prevent getting HIV. Just like women can take birth control, one pill every day to prevent getting pregnant, men can also take PrEP, one pill every day to prevent getting HIV. PrEP was approved by the FDA in 2012 and research has found that it can be very effective at preventing a person from getting HIV.” Young Black men who have sex with men were prompted through experiential questioning to discuss difficult thoughts, emotions, associations, memories, and sensations (TEAMS) that arise when considering attending a PrEP appointment. Based on the data collected from staff and young Black men who have sex with men interviews, we assessed the priorities for intervention content, the best methods for intervention delivery, and methods for recruitment and retention. This helped the research team identify and make needed adaptations to the ACTPrEP intervention material.

#### Interview Guide Sample Questions

Sample questions for the interview guide are as follows:

- Identifying values related to PrEP use: “Health: Some examples of values within health include: living a long life or being healthy enough to be physically active. What are some of your values within health? [Allow time to respond] How might these values relate to PrEP use?”
- Methods for delivering ACTPrEP: “Who would be the best person to provide the PrEP intervention?” “Where would be the best location to offer the PrEP intervention?”
- Techniques to cope with TEAMS: “As part of the intervention that we are developing we will be talking about different coping techniques. I am going to walk you through one of those exercises and get feedback from you. Choice in HIV Risk: Carrying Weight Metaphor Exercise: There are many variables that play into someone being at risk for HIV. Imagine you were born with a 500 pound weight on our shoulders and another person was born with 50 pounds of weight on their shoulders and you two were in a competition. The rules of the game are simple—the last one to sit down in their chair wins. Who do you think would win? Why? Exactly—this game isn’t fair and although you could technically ‘win’ the game would be much harder for you. This is very similar to individuals who are at higher risk for acquiring HIV. What if I offered to take 450 pounds off of your shoulder to make the game even—would you take me up on my offer? PrEP is similar to being offered the opportunity to take ‘weight’ off your shoulders and reduce your risk of HIV. It doesn’t mean that you are not in control of the behavior but it means that you are not in control of the amount of ‘weight’ you have to experience in order to reduce HIV risk.”

#### Analysis

All interviews with staff and young Black men who have sex with men were audio-recorded and transcribed by an outside HIPAA-certified transcription company. Transcripts were subsequently reviewed for accuracy by study research staff. A coding scheme was developed a priori based on the interview guide and previous research. To ensure interrater reliability, transcripts were coded independently by two members of the research team. Regular coding meetings were held to discuss and resolve coding discrepancies, make necessary edits to the coding scheme, and identify emerging themes. Coded qualitative data were organized using NVivo (Lumivero) software [30], which aided thematic analysis.

The developmental phase of the study took place between October 2021 and April 2022, during which 20 PrEP-eligible young Black men who have sex with men and 10 clinic staff who work directly with young Black men who have sex with men were interviewed. Among the young Black men who have sex with men interviewed, half were currently taking PrEP, and more than two-thirds had taken PrEP in the past. The length of experience clinic staff participants had working directly with young Black men who have sex with men widely varied, from less than 1 year to over 10 years.

The Adaptome implementation adaptation model was used to guide the interview content and assess which implementation adaptations were needed for ACTPrEP [31]. This model systematically helps define the core elements of an intervention and collects information regarding the different ways evidence-based interventions can be adapted to be delivered for diverse populations and contexts [32,33]. The Adaptome model organizes the adaptations by service setting, target audience, mode of delivery, and cultural considerations. Within service setting adaptations, participants noted that the interventionist should be PrEP-informed and well-integrated in the clinic. Participants also noted that ACTPrEP should be delivered in a variety of convenient locations. Within target audience adaptations, participants reported that the interventionist should provide check-in calls, be caring, and be able to authentically relate to young Black men who have sex with men’s lives. Within the mode of delivery adaptions, participants reported that the delivery of ACTPrEP should be flexible (number of sessions tailored to the participant, brief sessions, flexible scheduling, and option for web-based or in-person visits). Within cultural adaptations, participants noted that PrEP should be rebranded for all people and not just young Black men who have sex with men and PrEP should be compared to other health behaviors. These results were used to adapt ACTPrEP for successful implementation to young Black men who have sex with men living in Mississippi. Researchers were able to accommodate many of these adaptations in the final ACTPrEP protocol. Additionally, the values related to PrEP were in the life areas of relationships, health, intimacy, and life longevity. These values were used to tailor intervention content and as probes for the values section of the intervention. More details on the developmental phase findings can be found in another published study [34]. Notably, there were no significant differences in the responses provided by young Black men who have sex with men and staff [34].

#### Final ACTPrEP and ESOC Design

The final ACTPrEP core content elements include knowledge and beliefs about PrEP, identifying life values, self-compassion, cost of trying to control emotions, alternative perspective taking (different ways to think about PrEP), TEAMS related to taking HIV and PrEP, techniques used to help cope with the TEAMS related to taking PrEP (present moment awareness, tolerance of emotions, and actions consistent with one’s values), and structural barriers and facilitators for PrEP use. The final core content elements of the ESOC include knowledge and beliefs about PrEP and structural barriers and facilitators for PrEP use. The interventionist for both groups must have no prior professional training in providing mental health treatment. Study appointments may be delivered in a secure clinical space at one of the study sites or remotely via a HIPAA-compliant videoconferencing platform. There are 3 study sessions spaced 4 weeks apart (initial, 4 weeks, and 8 weeks). The first session will last approximately 45-60 minutes. The second and third sessions will last approximately 30 minutes. All intervention sessions will be audiotaped and uploaded to a secure file location on a secure server. The recordings will be used for fidelity checks. Participants will also complete a survey at three different times: initial visit, 12 weeks later, and 24 weeks later. Participants already taking PrEP at enrollment will also complete an adherence assessment using dried blood spot sampling and an adherence survey at baseline, 12 weeks, and 24 weeks. Participants who initiated PrEP after study enrollment will complete 2 additional study visits, including dried blood spot adherence assessment and adherence survey completion at 12 weeks, and 24 weeks post starting PrEP.

#### Pilot RCT Phase

Convenience sampling will be used to recruit 66 participants. Recruitment will occur in person through clinic staff and through flyer advertisements at several community clinics. Clinic staff will approach young Black men who have sex with men who appear to meet the inclusion criteria during their clinic visit and inquire about their interest in learning more about a research study on PrEP. Specifically, clinic staff will approach young Black men within the age range who are attending a PrEP appointment or who are presenting for sexually transmitted infection testing. Contact information for patients who are interested will then be shared with the research assistant. The research assistant will then reach out to these patients to screen them via phone or in person if they are able to meet with them in the clinic. Patients who are interested via the study flyer will reach out directly to the research assistant via phone. All interested patients will be screened for eligibility, and if eligible to participate, will be consented to through a HIPAA-complaint electronic signature platform (DocuSign) and scheduled for a session meeting. The consent will be completed in a private office or remotely, depending on the participants’ preference. Upon enrollment, participants will be randomized to receive either the intervention or ESOC.

#### ACTPrEP

The intervention arm will receive ACTPrEP, a manualized intervention based on ACT delivered by a trained PrEP navigator. A research assistant will be trained to deliver ACTPrEP through didactic instruction, role plays, and vignettes. All meetings will occur in person, on the phone, or via telehealth, depending on participant preference. ACTPrEP consists of one 60-minute initial session and two 30- to 45-minute follow-up sessions 4 weeks and 8 weeks later. The initial session will include the following discussion topics: values exercise, PrEP education, PrEP alternative perspective exercise, their control in reducing risk of acquiring HIV, barriers to PrEP use, self-compassion exercise, and an action plan. Session 2 will include the following discussion topics: a review of previous session, different forms of PrEP and side effects, problems with trying to control emotions, the cost of avoiding emotions, how to cope with emotions, and an action plan. Session 3 will include the following discussion topics: a review of the previous session, dimensions of sexuality, identifying influential people, helping self-prior to helping others, alternatives to controlling thoughts and emotions, exploring self-worth, perspective-taking exercise, and an action plan. The interventionist will receive ongoing supervision from the study team to promote fidelity throughout the trial. All sessions will be auto-recorded to assess for fidelity. A nonbiased researcher will be recruited to listen to the recordings and measure the fidelity. Participants will receive US $50 for each session that they complete.

#### The ESOC Arm

The control arm will receive ESOC, which will consist of one 30- to 45-minute initial session and two 30-minute follow-up sessions 4 weeks and 8 weeks later. All meetings will occur in person, on the phone, or via telehealth depending on participant preference. The initial session will include the following discussion topics: self-care, PrEP education, rates of HIV, structural barriers to PrEP use, and session summary. Sessions 2 and 3 will include the following discussion topics: self-care discussion, review of the previous session, consideration of PrEP, review of barriers, and session summary. All sessions will be auto-recorded to assess that no ACTPrEP intervention components were delivered to the control group. A nonbiased researcher will be recruited to listen to the recordings and measure this concept. Participants will receive US $50 for each session that they complete.

#### Assessments

Assessments will be administered at the initial visits and at 12 and 24 weeks to participants in both study arms. Each assessment will consist of an electronic survey via a HIPAA-complaint data collection platform (REDCap), which may take approximately 30-45 minutes to complete. Participants will receive US $30 for each assessment that they complete.

#### Dried Blood Spot

If participants are taking PrEP or decide to get a prescription for PrEP, they will complete additional visits for dried blood spots. This will measure participant adherence to PrEP. If a participant is enrolled in the study while already taking PrEP, they will have a dried blood spot collected at the initial visit and at 12 weeks and 24 weeks. If a participant is enrolled in the study while not already taking PrEP, they will have a dried blood spot collected at 12 weeks and 24 weeks after they pick up their prescription from the pharmacy. Each dried blood spot visit will include a 5-minute electronic survey via a HIPAA-complaint data collection platform (REDCap) to assess self-report PrEP adherence. Participants will be compensated US $30 after each of the dried blood spot visits they complete.

#### Analysis

Data analysis will be conducted using SPSS (IBM Corp) or SAS (SAS Institute) statistical software. All analyses will be conducted according to a predefined statistical analysis plan to ensure transparency and reproducibility. Following data collection, intervention content with mean Client Satisfaction Questionnaire scores below 24 will be reviewed for potential revisions. Additionally, qualitative interviews will be conducted with a purposive sample of participants (both engaged and not engaged in PrEP) and PrEP navigators to explore perceived barriers and facilitators to implementing ACTPrEP within the clinical setting. This information will be used to refine the ACTPrEP intervention for a larger RCT.

#### Descriptive Statistics and Baseline Group Comparisons

Descriptive statistics (means, SDs, and frequencies) will be calculated to summarize participant characteristics at baseline. This will provide an overview of the sample demographics (age, race, and education), sexual behavior (number of sexual partners and condom use), and baseline knowledge and attitudes about PrEP. Between-group differences in baseline characteristics (ACTPrEP vs ESOC) will be assessed using appropriate statistical tests. For continuous variables, such as age or number of sexual partners, independent sample 2-tailed *t* tests will be used. Categorical variables, like race or education level, will be compared using chi-square tests. If the assumptions for chi-square tests are not met, nonparametric tests like the Mann-Whitney *U* test will be used.

#### Feasibility and Acceptability Outcomes

We will calculate rates (proportions) for each feasibility measure, including eligibility, consent, randomization, retention (across all assessments), and session attendance. This will provide a clear picture of recruitment, retention, and intervention delivery within the pilot study. The Client Satisfaction Questionnaire scores will be averaged to assess the overall acceptability of the ACTPrEP intervention. Scores lower than 24 will be flagged for further review to identify areas for improvement in the intervention content.

#### PrEP Use and Adherence

We will evaluate differences in the proportion of participants who engage in PrEP across the 2 groups (ACTPrEP vs ESOC). This will be determined by reviewing patients’ medical records and pharmacy data to identify those who attended a PrEP medical visit or filled a PrEP prescription. While not powered to definitively assess these outcomes, descriptive statistics will be generated for adherence and persistence measures (eg, self-reported adherence and pharmacy refill data). This will provide preliminary data to inform future studies.

#### Secondary Outcomes

Descriptive statistics will also be summarized for secondary outcomes, including PrEP knowledge, attitudes toward PrEP care, and HIV risk behavior scores. Changes in these measures over time will be explored descriptively.

#### Generalized Linear Models

Data will be aggregated across follow-up assessments (baseline, 6 weeks, and 12 weeks) and analyzed using generalized linear models to assess the intervention effect on continuous secondary outcomes (eg, PrEP knowledge scores). This will allow us to examine differences in the linear change over time between the ACTPrEP and ESOC groups on each outcome variable. Propensity scores will be used to account for any potential baseline imbalances in characteristics between the 2 groups (ACTPrEP vs ESOC). This will help to strengthen the causal inferences drawn from the intervention effect.

#### Power Analysis and Missing Data

With the primary purpose of this pilot study being to examine feasibility and acceptability, we recognize that the power to detect statistically significant differences between groups on secondary outcomes may be limited. However, the power analysis conducted with a conservative estimate of retention (85%) provided preliminary data to inform future studies. We anticipate a maximum of 15% missing data due to attrition or incomplete responses. To address missing data, multiple imputations by chained equations will be used. This technique creates multiple imputed datasets based on available data and model-based assumptions, allowing for the inclusion of participants with missing values in the analyses.

### Ethical Considerations

All procedures performed in studies involving human participants were in accordance with the ethical standards of the institutional and national research committee and with the 1964 Helsinki Declaration and its later amendments or comparable ethical standards. The study was reviewed and approved by the University of Mississippi Medical Center institutional review board, and its conduct was consistent with applicable federal law (FWA00003630). All participants complete written informed consent prior to participating. All study data were deidentified. All participants receive US $50 in cash or a gift card for each completed study session, US $30 for each completed survey, and US $30 for each completed dried blood spot appointment.

## Results

The RCT phase of this study is ongoing, with initial institutional review board approval obtained in March 2023. Research activities commenced in September 2023 and are expected to continue through June 2026. As of February 2025, ten participants have been enrolled in the pilot RCT phase. Given the early stage of the trial, we are not yet able to report on the intervention’s impact on the desired outcomes. Preliminary findings are anticipated to be available in late 2026 (Table 1).

**Table 1:** Pilot RCT^a^ timeline.

Quarters	2023					2024					2025					2026			
	1	2	3	4	1		2	3	4	1		2	3	4	1		2	3	4
																
Recruitment (RCT)				✓	✓		✓	✓	✓	✓		✓	✓	✓	✓		✓		
Randomized pilot				✓	✓		✓	✓	✓	✓		✓	✓	✓	✓		✓	✓	
Analyses													✓	✓	✓		✓	✓	
Publish final RCT manuscript																		✓	✓

^a^RCT: randomized controlled trial.

## Discussion

Despite the high efficacy of PrEP in preventing HIV infection, significant racial and ethnic disparities persist in PrEP uptake and adherence [8-10]. Young Black men who have sex with men are particularly vulnerable to HIV infection yet encounter numerous structural, psychosocial, and systemic barriers that hinder PrEP use [11-13]. These barriers include stigma, medical mistrust, cost concerns, limited health care access, and insufficient knowledge about PrEP [11-13]. Addressing these disparities is critical to ending the HIV epidemic in the United States [35]. This pilot RCT is designed to assess the feasibility and acceptability of ACTPrEP, an intervention based on ACT, for promoting PrEP use among young Black men who have sex with men. A pilot RCT is an important first step for evaluating an adapted intervention and assessing best implementation practices for a specific population. It allows us to refine the intervention based on participant feedback, assess recruitment and retention strategies, and determine the feasibility of a larger-scale RCT to evaluate efficacy.

This study builds on the breadth of prior research establishing the effectiveness of ACT-based interventions for behavior change in various health domains, including substance use, smoking cessation, and mental health treatment [21-27]. Emerging literature supports the use of ACT in HIV prevention, particularly for addressing psychological barriers to PrEP adherence, such as stigma, avoidance behaviors, and internalized HIV-related fears [34,36,37]. Studies have also shown that culturally tailored, theoretically driven behavioral interventions can improve PrEP use among Black men who have sex with men [38,39]. By integrating ACT principles, ACTPrEP uniquely targets psychological flexibility, a construct that has been associated with improved health behaviors and medication adherence. Many existing approaches focus on practical barriers (eg, cost, transportation, and insurance navigation), whereas ACTPrEP takes a more comprehensive approach by addressing both psychological and structural obstacles to PrEP use. This study adds to the growing body of literature by evaluating a behavioral intervention tailored specifically for young Black men who have sex with men, a population that remains underrepresented in PrEP research.

The ACTPrEP intervention leverages ACT principles to help young Black men who have sex with men identify and address psychological barriers that may impede PrEP uptake and adherence. The control arm receives ESOC consisting of general PrEP information, a video, and support for overcoming logistical barriers such as transportation and cost. This study has several strengths. It focuses on a population with a high burden of HIV and a significant unmet need for PrEP. The intervention is theoretically grounded in ACT, a well-established intervention for behavior change. While this study offers valuable insights, several limitations must also be acknowledged. Since ACTPrEP focuses on both PrEP update and PrEP persistence, to enhance recruitment the inclusion criteria were slightly changed to include both young Black men who have sex with men taking PrEP and those not taking PrEP. Participants taking PrEP may have offered different recommendations during the developmental phase of the study. As a pilot RCT, the sample size is limited, restricting generalizability to broader populations of young Black men who have sex with men. Future studies should increase sample size to enhance statistical power and allow for subgroup analyses. The intervention is being tested at a single site, which may not capture the full diversity of experiences and structural barriers faced by young Black men who have sex with men across different geographic regions. Multisite trials in diverse locations are necessary to assess broader implementation potential. A larger-scale trial should also include longer follow-up periods to assess sustained PrEP adherence.

If findings from this pilot study demonstrate that ACTPrEP is feasible and acceptable, we will refine ACTPrEP based on participant and provider feedback and scale it up in a larger multisite RCT to evaluate both effectiveness in improving PrEP uptake and adherence. Given the urgent need for culturally tailored PrEP interventions for young Black men who have sex with men, ACTPrEP has the potential to be integrated into numerous HIV prevention services offered by community-based organizations, sexual health clinics, and PrEP navigation programs. Future directions also include testing different implementation strategies to enhance scalability and accessibility.

The ongoing HIV epidemic among young Black men who have sex with men necessitates effective interventions to promote PrEP use. ACT has the potential to address psychological barriers that hinder PrEP uptake and adherence. This pilot RCT will provide valuable data on the feasibility and acceptability of ACTPrEP as a novel intervention for increasing PrEP use among young Black men who have sex with men. Based on the findings of this pilot study, we may refine the intervention and conduct a larger-scale RCT to assess its efficacy in promoting PrEP use in this high-risk population.

## Abbreviations

ACT: acceptance and commitment therapy
ESOC: Enhanced Standard of Care
HIPAA: Health Insurance Portability and Accountability Act
PrEP: pre-exposure prophylaxis
RCT: randomized controlled trial
REDCap: Research Electronic Data Capture
TEAMS: thoughts, emotions, associations, memories, sensations

## Data Availability

The qualitative datasets generated or analyzed during this study are not publicly available given the sensitive nature of the qualitative interviews but are available from the corresponding author on reasonable request. Data from the pilot randomized controlled trial will become publicly available once all data have been collected, which is anticipated by June 2026.
